# The number of displaced rib fractures is more predictive for complications in chest trauma patients

**DOI:** 10.1186/s13049-017-0368-y

**Published:** 2017-02-28

**Authors:** Chih-Ying Chien, Yu-Hsien Chen, Shih-Tsung Han, Gerald N. Blaney, Ting-Shuo Huang, Kuan-Fu Chen

**Affiliations:** 1 Department of General Surgery, Chang Gung Memorial Hospital, Keelung, Taiwan; 2 Department of Emergency Medicine, Chang Gung Memorial Hospital, Linkou, Taiwan; 3 Department of Emergency Medicine, Chang Gung Memorial Hospital, Keelung, Taiwan; 4 Clinical Informatics and Medical Statistics Research Center, Chang Gung University, Taoyuan, Taiwan; 5 Community Medicine Research Center, Chang Gung Memorial Hospital, Keelung, Taiwan

**Keywords:** Rib Fractures, Displaced Rib Fractures, Complications, Chest Trauma, Prognosis, Sensitivity and Specificity, Hospitalization

## Abstract

**Background:**

Traumatic rib fractures can cause chest complications that need further treatment and hospitalization. We hypothesized that an increase in the number of displaced rib fractures will be accompanied by an increase in chest complications.

**Methods:**

We retrospectively reviewed the trauma registry between January 2013 and May 2015 in a teaching hospital in northeastern Taiwan. Patients admitted with chest trauma and rib fractures without concomitant severe brain, splenic, pelvic or liver injuries were included. The demographic data, such as gender, age, the index of coexistence disease, alcohol consumption, trauma mechanisms were analyzed as potential predictors of pulmonary complications. Pulmonary complications were defined as pneumothorax, hemothorax, flail chest, pulmonary contusion, and pneumonia.

**Results:**

In the 29 months of the study period, a total of 3151 trauma patients were admitted to our hospital. Among them, 174 patients were enrolled for final analysis. The most common trauma mechanism was road traffic accidents (58.6%), mainly motorbike accidents (*n* = 70, 40.2%). Three or more displaced rib fractures had higher specificity for predicting complications, compared to three or more total rib fractures (95.5% vs 59.1%). Adjusting the severity of chest trauma using TTSS and Ribscore by multivariable logistic regression analysis, we found that three or more rib fractures or any displaced rib fracture was the most significant predictor for developing pulmonary complication (aOR: 5.49 95% CI: 1.82–16.55). Furthermore, there were 18/57 (31.6%) patients with fewer than three ribs fractures developed pulmonary complications. In these 18 patients, only five patients had delayed onset complications and four of them had at least one displaced rib fracture.

**Discussion:**

In this retrospective cohort study, we found that the number of displaced or total rib fractures, bilateral rib fractures, and rib fractures in more than two areas were associated with the more chest complications. Furthermore, three or more rib fracture or any displacement were found to be the most sensitive risk factor for chest complications, independent of other risk factors or severity index.

**Conclusion:**

The number of displaced rib fractures could be a strong predictor for developing pulmonary complications. For patients with fewer than three rib fractures without rib displacement and initial lung or other organ injuries, outpatient management could be safe and efficient.

**Electronic supplementary material:**

The online version of this article (doi:10.1186/s13049-017-0368-y) contains supplementary material, which is available to authorized users.

## Background

Chest trauma comprises 10–15% of all trauma cases [1]. Chest trauma is mostly due to blunt injury, including road traffic accidents and falls, and about one-third of cases occur with rib fractures [2]. The risk of severe morbidity and mortality increases as the number of rib fractures increases [3, 4]. A systematic review and meta-analysis showed a pooled odds ratio of 2.02 (95% CI: 1.89–2.15) for mortality in patients with three or more rib fractures, suggesting that hospitalization should be warranted for those patients [5]. Fractured ribs sometimes are displaced and could penetrate the pleura, causing lung parenchyma injury. It has been reported that patients with displaced rib fractures are prone to develop morbidities such as delayed hemothorax [6]. Studies investigating the relationship between complication rates and the number of displaced rib fractures, however, are still lacking.

Several scoring systems were developed to assist prediction-based decision-making based on clinical outcomes. Ribscore is a pure anatomic scoring system to predict outcomes such as pneumonia, respiratory failure, and tracheostomy. Ribscore suggests the numbers of displaced rib fracture as one of the important risk factors [7]. Thoracic trauma severity score (TTSS) was developed for prediction of outcome such as mortality and acute respiratory distress syndrome (ARDS) [8, 9]. However, because these studies, did not exclude patients with multiple severe organ injuries not directly related to chest trauma, the performance of these scoring systems among patients with minor chest trauma still remains unclear.

This retrospective cohort study enrolled patients with chest trauma without multiple severe organ injuries and utilized the prospectively recorded trauma registry, electronic medical records, and radiographic images. We hypothesized that the more displaced rib fractures in patients with chest trauma, the more accompanied chest complications will ensue. We also investigated factors that could predict the complications of rib fractures.

## Methods

We retrospectively reviewed the trauma registry and medical records between January 2013 and May 2015 at the Chang Gung Memorial Hospital, Keelung, Taiwan. Our hospital is a teaching hospital with 1089 beds and is categorized as an advanced emergency responsibilities hospital by the Ministry of Health and Welfare. There are about 1300 trauma patients admitted to our hospital every year and about one-fifth are referrals. All patients were treated according to the Advanced Trauma Life Support (ATLS) guideline [10]. Whole body computed tomography (CT) was arranged if the patients suffered from major trauma mechanisms (high-speed motor vehicle accident, pedestrian run over by a vehicle, thrown from car, etc.) and had no absolute contraindication. Minor chest trauma patients would be arranged chest radiograph (CXR) and rib view radiograph for the initial survey. Chest CT would also be arranged for patients with a positive chest injury finding (e.g. flail chest, multiple rib fractures, and lung contusion) and for those who needed mechanical ventilation due to receiving surgical interventions. Patients with flail chest, multiple rib fractures, respiratory distress, and unstable vital signs were admitted to the intensive care unit (ICU). The surgical indications for emergent thoracotomy include massive hemothorax with evacuation of more than 1500 mL of blood immediately after tube thoracostomy and persistent bleeding from the chest with 200 cc per hour for consecutive three hours. The surgical indications for video-assisted thoracoscopic surgery (VATS) included retained hemothorax after tube thoracostomy for 3–7 days and pneumothorax with persistent air leak for 3 days after tube thoracostomy. One dedicated trauma registrar recorded all demographic data such as gender, age, index of coexistence disease (ICED) and alcohol consumption [11] of trauma patients in this trauma databank. The records include demographic data, pre- and post-hospital status, trauma mechanisms such as a motorbike, car, bicycle, pedestrian, fall, and others, interventions throughout the emergency department and hospital course, and outcomes such as length of stay and in-hospital mortality.

Two-step inclusion method was adopted in this study: 1) hospitalized patients with evidence of rib fracture were eligible for inclusion review; 2) patients with concomitant severe brain injury, severe splenic injury, severe pelvic injury or severe liver injury (defined as AIS≧4) were excluded for final analysis to simplify potential causal relationship between rib fracture and outcomes. Diagnosis of rib fracture was made by standard CXR, rib view radiograph, or chest CT. All CXR, rib view radiograph, and chest CT were re-examined by the first and the second author separately, and discrepant results were further resolved by consensus meetings. The numbers of displaced and non-displaced rib fractures were recorded separately after the meetings. A displaced rib fracture was defined as a displacement distance at least half of the rib width (Additional file 1: Figure S1a and b). The locations of rib fractures were classified into upper (1st–3rd), middle (4th–9th), and lower (10th–12th). The laterality of rib fractures was recorded as the right side, left side and both sides. The injury severity score (ISS) [12] and abbreviated injury scale (AIS) [13] were also recorded.

Our major outcomes were pulmonary complications, defined as pneumothorax, hemothorax, flail chest, pulmonary contusion, or pneumonia. Secondary outcomes included respiratory failure defined as on ventilator for more than 48 h, tube thoracostomy, and delayed onset complications defined as pneumohemothorax, pneumonia, and pulmonary contusion becoming evident until 24 h after admission.

We calculated RibScore and TTSS for each patient in our study. The Ribscore was the sum of six 1-point variables: (1) six or more rib fractures (2) bilateral fractures (3) flail chest (4) three or more severely displaced fractures (5) first rib fracture and (6) at least one fracture in all three anatomic areas (anterior, lateral, and posterior) [7]. According to the Ribscore, we categorized all patients into three groups: 0–1, 2–3 and 4–6. The TTSS incorporates age, respiratory status, rib fracture numbers, pulmonary contusion and pleural involvement on admission and we categorized the patients according to the TTSS into four groups: 0–4, 5–7, 8–12, and 13–25 [14].

As to the statistical methods, we used Pearson’s x^2^ test and Fisher’s exact test to distinguish the potential risk factors. Statistical significance was defined as a two-tail *p-*value less than 0.05. Sensitivity, specificity, positive predictive value (PPV) , negative predictive value (NPV), and the 95% confidence interval were calculated to find the recommended cut-off values for clinical utility. We combined the numbers of total and displaced rib fractures to find the best cut-off value. Multivariable logistic regression models were applied to evaluate the performance of the numbers of total and displaced rib fracture adjusting for the severity of the chest trauma using TTSS and Ribscore. Statistical analysis was performed by SPSS (version 17.0; SPSS, Chicago, IL, USA).

We followed the Strengthening the Reporting of Observational Studies in Epidemiology (STROBE) statement to report this observational study [15]. The institutional review board approved this study (104–7586B) and waived the informed consents owing to the retrospective nature of this study.

## Results

**Fig. 1:**
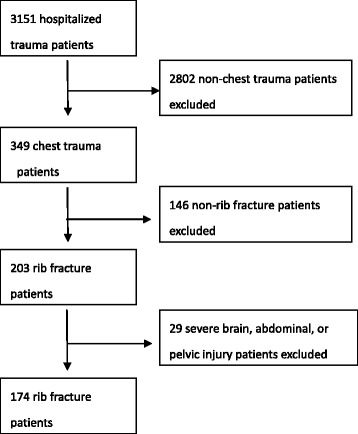
Flow chart of study

A total of 3151 patients with trauma were admitted to our hospital during the 29 months of the study period. Chest trauma (*n* = 349) comprised 11.1% of the total hospitalization and among them, 203 patients had traumatic rib fractures (6.4% of the total hospitalization). After excluding 29 patients with concomitant severe brain injury, severe splenic injury, severe pelvic injury or severe liver injury (14%), 174 patients remained for final analysis (Fig. 1 and Table 1). Among those enrolled patients, most were males (*n* = 121, 69.5%) and younger than 65 years old (*n* = 131, 75.3%). The mechanisms of trauma included road traffic accidents (58.6%), falls (39.7%), assault (0.6%), and compression by heavy objects (1.1%). The most common road traffic accident was motorbike accident (40.2%), followed by car accident (7.5%). Fall was more prevalent in the elderly group (age≧65 year, 51.2% vs.35.9%, *p* = 0.056). The average ISS score was 14.4. The average ICU length of stay was 5.97 days. All the patients received CXR examination and chest CT was performed in 61.5% patients. Isolated rib fractures without any other organ injury were found in 75 patients (43.1%).

**Table 1:** Demographic data

Parameter	Total (*N* = 174)	Patients with pulmonary complication (*N* = 108)	OR 95% CI	*p*-value
	[n (%)]	[n (%)]		
Gender				
Male	121 (69.5)	75 (62)	0.99 (0.51–1.92)	0.97
Elderly				
≧65	43 (24.7)	26 (60.5)	0.91 (0.45–1.85)	0.80
ICED				
0	69 (39.7)	46 (66.7)	Reference	-
1	88 (50.6)	53 (60.2)	0.76 (0.39–1.46)	0.41
2	17 (9.8)	9 (52.9)	0.56 (0.19–1.65)	0.30
3	0		NA	NA
Mechanism				
Motorbike	70 (40.2)	39 (55.7)	Reference	-
Car	13 (7.5)	10 (76.9)	2.65 (0.67–10.47)	0.16
Bicycle	8 (4.6)	5 (62.5)	1.33 (0.29–5.98)	0.72
Pedestrian	11 (6.3)	7 (63.6)	1.39 (0.37–5.19)	0.62
Fall ≧3 m	29 (16.7)	21 (72.4)	2.09 (0.81–5.35)	0.13
Fall <3 m	40 (23)	23 (57.5)	1.08 (0.49–2.36)	0.86
Assault	1 (0.6)	1 (100)	NA	NA
Heavy stuff compression	2 (1.1)	2 (100)	NA	NA
Laterality				
Left	91 (52.3)	57 (62.6)	Reference	-
Right	71 (40.8)	39 (54.9)	0.73 (0.39–1.37)	0.32
Both	12 (6.9)	12 (100)	NA	NA
Location				
1st–3rd	12 (6.9)	7 (58.3)	Reference	-
4th–9th	117 (67.2)	66 (56.4)	0.89 (0.27–2.98)	0.85
10th–12th	10 (5.7)	6 (60)	1.07 (0.19–5.91)	0.94
≧2area	35 (20.1)	29 (82.9)	4.29 (0.97–18.97)	0.06
Rib fracture numbers				
1–2	57 (32.8)	18 (31.6)	Reference	-
≧3	117 (67.2)	90 (76.9)	7.22 (3.57–14.62)	<0.0001
Displaced rib fracture numbers				
0	40 (23)	13 (32.5)	Reference	-
≧1	134 (77)	95 (70.9)	5.06 (2.37–10.81)	<0.0001
Nondisplaced rib fracture numbers				
0	33 (19)	21 (63.6)	Reference	-
≧1	141 (81)	87 (61.7)	0.92 (0.42–2.02)	0.837
Alcohol drinking	13 (7.5)	8 (61.5)	0.98 (0.31–3.12)	0.97
ISS				
≧16	88 (50.6)	81 (92)	25.29 (10.32–61.97)	<0.0001
< 16	86 (49.4)	27 (31.4)	Reference	-
AIS (thoracic area)				
≧4	71 (40.8)	70 (98.6)	NA	NA
< 4	103 (59.2)	38 (36.9)		
Ribscore				
0–1	138 (79.3)	73 (52.9)	Reference	-
2–3	28 (16.1)	27 (96.4)	24.04 (3.18–181.91)	0.002
4–6	8 (4.6)	8 (100)	NA	NA
TTSS				
0–4	66 (37.9)	22 (33.3)	Reference	-
5–7	67 (38.5)	45 (67.2)	4.09 (1.99–8.43)	0.0001
8–12	30 (17.2)	30 (100)	NA	NA
13–25	11 (6.3)	11 (100)	NA	NA

Among those 108 patients (62.1%) developing pulmonary complications, 99 patients had pneumothorax or hemothorax, in whom 53 patients recovered after conservative treatment and 46 patients needed further interventions including chest tube insertion and pigtail tube insertion (Table 2). Four patients (3.7%) received surgical treatments. Those surgical procedures included VATS for evacuation of retained hemothorax, thoracoscopic wedge resection of left upper lobe for laceration with bleeding and flail chest rib fixation. In four mortality cases (2.3%), two were due to severe desaturation by pulmonary contusion and hemopneumothorax, one was due to severe pulmonary contusion and persistent desaturation even with full ventilator support, and another was due to massive hemothorax.

**Table 2:** Outcome list

Outcome	Total (*N* = 174)
Pulmonary complications	108 (62.1)
Pneumothorax	53 (30.5)
Hemothorax	82 (47.1)
Pulmonary contusion	52 (29.9)
Flail chest	5 (2.9)
Respiratory failure	17 (9.8)
Tube thoracostomy	46 (26.4)
Surgical intervention	4 (2.3)
Mortality	4 (2.3)
ICU admission	40 (23)
Delayed complications	33 (19)

### Location of rib fracture and complication

There was no statistically significant difference in regard to the complication rates between right or left side rib fractures (right: 62.6% vs. left: 54.9%). However, if both side rib fractures were occured, the complication rate was much higher (*n* = 12, 100%, *p* = 0.02). The mechanisms of bilateral rib fractures included motorbike accident (*n* = 4), falling from the height (*n* = 4), pedestrian accident (*n* = 3), and compression by heavy objects (*n* = 1). The most common location of rib fractures was middle (4–9th, 67.2%). There was no statistically significant difference between fracture location and complication rate (upper: 58.3%, middle: 56.4%, lower: 60%). However, if rib fractures occurred at more than two locations, the complication rate increased (*n* = 29, 82.9%, *p* = 0.06).

### Number of rib fracture and complication

**Fig. 2:**
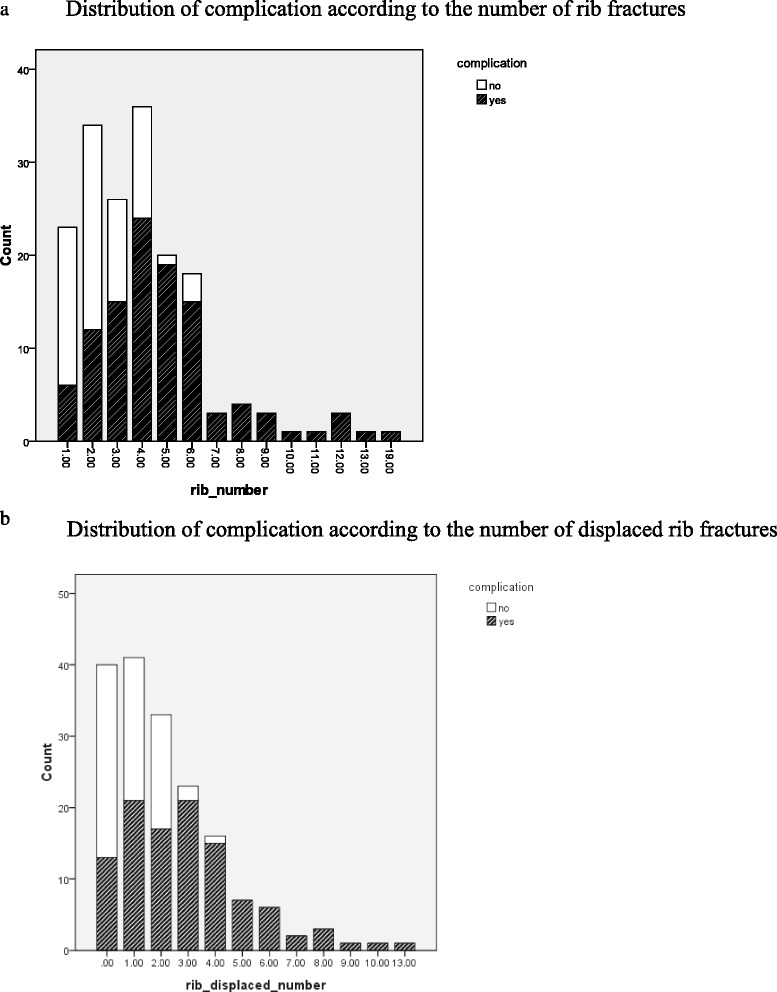
**a** Distribution of complication according to the number of rib fractures. **b** Distribution of complication according to the number of displaced rib fractures

We further found that complication rates increased in correlation with the number of total and displaced rib fractures (Tables 3 and 4). The commonly used number of three or more rib fractures threshold had low specificity for predicting complications such as pneumothorax, hemothorax, and pulmonary contusion in our study (59.1%, 95% CI:46–71% Table 5). In contrast, the number of three or more displaced rib fractures had higher specificity and PPV in predicting complications (95.5% and 95%, respectively, Table 6). Among 66 patients (37.9%) having rib fractures without any pulmonary complication developed during their hospitalization, 27 patients had three or more rib fractures (40.9%. Fig. 2a) and only three patients had three or more displaced rib fractures (4.5%, Fig. 2b). For those 34 patients with only two rib fractures, 12 patients had pulmonary complications, and four patients experienced delayed complications. If we excluded one 20-year-old patient, the remaining three patients with delayed complications had at least one displaced rib fracture and two had tube thoracostomy intervention. There were 18/57 (31.6%) patients who had fewer than three ribs fractures and developed pulmonary complications. If the patients had fewer than three rib fractures, we found a 38.7% (12/31) complication rate in patients with one or two displaced rib fractures, compared to a 22.22% (6/27) complication rate in patients without any displaced rib fracture. Among the eight patients aged less than 20 years old, all developed pulmonary complications, including eight pulmonary contusions, four bilateral pulmonary contusions, and seven pneumothorax or hemothorax. Among those young patients, only one had fewer than three rib fractures, and three had no displaced rib fracture.

**Table 3:** The complication rates and the number of rib fractures

Number of rib fractures	Patients with Complication (complication rate)	Total number of patients
1	6 (26.1%)	23
2	12 (35.3%)	34
3	15 (57.7%)	26
4	24 (66.7%)	36
5	19 (95%)	20
6	15 (83.3%)	18
≧7	17 (100%)	17

**Table 4:** The complication rates and the number of displaced rib fractures

Number of displaced rib fractures	Patients with Complication (complication rate)	Total number of patients
1	21 (51.2%)	41
2	17 (51.5%)	33
3	21 (91.3%)	23
4	15 (93.8%)	16
5	7 (100%)	7
6	6 (100%)	6
≧7	8 (100%)	8

**Table 5:** The correlation between numbers of rib fracture and complication

	Patient with complication	Patient without complication	Sensitivity (95% CI )	Specificity (95% CI )	PPV (95% CI )	NPV (95% CI )	OR (95% CI)
Rib fx ≧2	102	49	94.5% (88–98%)	25.8% (16–38%)	67.6% (59–75%)	73.9% (52–90%)	5.90 (2.19–15.89%)
Rib fx≧3	90	27	83.3% (75–89%)	59.1% (46–71%)	76.9% (68–84%)	68.4% (55–80%)	7.22 (3.57–14.62%)
Rib fx≧4	75	16	69.4% (60–78%)	75.8% (64–85%)	82.4% (73–90%)	60.2% (49–71%)	7.10 (3.54–14.25%)
Rib fx≧5	51	4	47.2% (38–57%)	93.9% (85–98%)	92.7% (82–98%)	52.1% (43–61%)	13.87 (4.71–40.81%)
Total	108	66					

*CI* confidence interval, *PPV* positive predictive value, *NPV* negative predictive value, *OR* odds ratio, *fx* fracture

**Table 6:** The correlation between the number of displaced rib fractures and complication

	Patient with complication	Patient without complication	Sensitivity (95% CI )	Specificity (95% CI )	PPV (95% CI )	NPV (95% CI )	OR (95% CI )
Displaced rib fx ≧1	134	40	88.0% (80–93%)	40.9% (29–54%)	70.9% (62–78%)	67.5% (51–81%)	5.06 (2.37–10.81%)
Displaced Rib fx≧2	74	19	68.5% (59–77%)	71.3% (59–82%)	79.6% (70–87%)	58% (47–69%)	5.38 (2.76–10.52%)
Displaced Rib fx≧3	57	3	52.8% (43–62%)	95.5% (87–99%)	95% (86–99%)	55.3% (46–65%)	23.47 (6.94–79.36%)
Displaced Rib fx≧4	36	1	33.3% (25–43%)	98.5% (92–100%)	97.3% (86–100%)	47.5% (39–56%)	32.50 (4.33–243.80%)
Displaced Rib fx≧5	21	0	19.4% (12–28%)	100% (95–100%)	100% (84–100%)	43.1% (35–51%)	32.68 (1.94–549.35%)
Total	108	66					

*CI* confidence interval, *PPV* positive predictive value, *NPV* negative predictive value, *OR* odds ratio, *fx* fracture

Furthermore, adjusting the severity of chest trauma using TTSS and Ribscore by multivariable logistic regression analysis, we also evaluated the performance of the numbers of total and displaced rib fracture to predict the pulmonary complication. We found that three or more rib fractures or any displaced rib fracture was the most significant predictor for developing pulmonary complication (aOR: 5.49 95% CI: 1.82–16.55, Table 7).

**Table 7:** Univariate and multivariate logistic regression analysis evaluating three or more total and any displaced rib fracture in predicting pulmonary complications

	Univariable			Multivariable		
	OR	CI	*p*-value	aOR	95% CI	*p*-value
Three or more rib fractures	7.22	3.57–14.62	0.0001	3.72	1.68–8.26	0.001
Any displaced rib fracture	5.06	2.37–10.81	0.0001	4.79	1.87–12.31	0.001
Three or more rib fractures or any displacement	7.93	3.00–20.99	0.0001	5.49	1.82–16.55	0.002

## Discussion

In this retrospective cohort study, we found that the numbers of displaced or total rib fractures, bilateral rib fractures, and rib fractures in more than two areas were associated with pulmonary complications including pneumothorax, hemothorax, and pulmonary contusion. For patients with fewer than three rib fractures without rib displacement and initial lung or other organ injuries, outpatient management could be safe and efficient.

Bilateral rib fractures were rare in our study (6.9%) but the complication rate greatly increased (100%). The exceptional force of dangerous mechanisms, including falling from the height or pedestrian run over by a car, could cause bilateral rib fractures. In the pedestrian group, 27.3% had bilateral rib fractures. As reported by Livingston et al., we also found that for patients with rib fractures located in more than one anatomic regions, the complication rate increased [16]. Bilateral rib fracture and rib fractures at multiple regions, therefore, could serve as good indicators for complications.

Many studies found a similar trend of increasing complication rate with the greater number of rib fractures as ours [3, 4]. Hospitalization, therefore, is warranted if three or more rib fractures are found. There were, however, 18/57 (31.6%) patients who had fewer than three ribs fractures developed pulmonary complications. In these 18 patients with pulmonary complications, five patients had delayed onset complications. Except one 20 year-old patient, the remaining four patients all had at least one displaced rib fracture. In other words, for patients with fewer than three rib fractures without rib displacement, delayed pulmonary complication was rare if initial examinations revealed no lung injury. Furthermore, our results indicate that three or more rib fractures has low specificity (59.1%) for predicting complications such as pneumothorax, hemothorax, pulmonary contusion, and flail chest (Table 5). Instead, three or more displaced rib fractures has higher specificity for predicting complications (95.5%, Table 6). Some case reports show that patients with displaced rib fractures are prone to develop delayed hemothorax [6]. Displaced rib fractures are more likely to cause intercostal vessel injuries and penetrate the pleura into lung parenchyma. The sensitivity of three or more rib fractures or any displacement to predict pulmonary complications is higher than TTSS and Ribscore (Table 8). Therefore, for patients with minor chest trauma, fewer than three rib fractures without displacement could be an indicator for safe and efficient outpatient management.

**Table 8:** The performance of three or more total and any displaced rib fracture, TTSS and Ribscore in prediction of complication

	Patient with complication	Patient without complication	Sensitivity (95% CI )	Specificity (95% CI )	PPV (95% CI )	NPV (95% CI )	OR (95% CI )
Three or more rib fracture or any displacement	102	45	94.4% (88–98%)	31.8% (21–44%)	69.4% (61–77%)	77.8% (58–91%)	7.93 (3–20.99)
TTSS > 4	86	22	79.6% (71–87%)	66.7% (54–78%)	79.6% (71–87%)	67% (54–78%)	7.82 (3.91–15.6)
TTSS > 7	41	0	38% (29–48%)	100% (95–100%)	100% (91–100%)	49.6% (41–58%)	NA
Ribscore >0	81	17	75% (66–83%)	74.2% (62–84%)	82.7% (74–90%)	64.5% (52.7–75.1%)	8.65 (4.28–17.46)
Ribscore > 1	35	1	32.4% (24–43%)	98.5% (92–100%)	97.2% (85–100%)	47.1% (39–56%)	31.16 (4.15–233.91)

Because of the increasing popularity of high-resolution CT, diagnosing rib fractures nowadays is more accurate than it was decades ago, and even tiny linear rib fracture can be detected . Furthermore, tiny pneumothorax and hemothorax are also easily detected as well. Simple non-displaced rib fracture detected by higher resolution CT may not have the much clinical significance. These factors could also contribute to the difference between total or displaced rib fractures in predicting severe comorbidities.

Patients with rib fractures need aggressive pain control, pulmonary toilet, adequate respiratory rehabilitation and judicious fluid management to prevent potential delayed pulmonary complications. Several pain control methods, such as subcutaneous local injection regional block and epidural catheter placement with opioids infusion, can be applied [17, 18]. However, if the risk is unrecognized by the health care providers, patients without appropriate treatment may develop complications such as atelectasis and pneumonia. Therefore, simple predictors of potential complications would be important to improve patient outcomes.

The mortality rate of chest trauma ranged from 5.7% to 12% [3, 19–21]. In the present study, the mortality rate was 2.4%, which is probably due to the exclusion of patients with severe brain, liver, spleen, or pelvic injuries. Avila Martinez et al. also noted a low mortality rate of 1.1% when severe brain injury, abdominal injury and ventilator usage were excluded [22].

Our study has several limitations. First, the sample size and mortality rate in our study are small. However, we used several different stratified analyses to overcome this limitation. Second, not all patients received chest CT. The accuracy, and especially sensitivity, of the number of rib fractures to detect pneumothorax and hemothorax could be over-estimated by verification bias. Briefly, patients who were suspected of having pneumothorax and hemothorax, either by physical examination or plain radiograph, would disproportionally more likely to receive more chest CT, and therefore more minor or linear rib fractures might be found. However, if we only select the population of patients who must receive chest CT, the population may lack the minor rib fractures group. Furthermore, the main mechanism of chest trauma of our study population is motorbike accident and we caution readers to generalize our results to other populations.

## Conclusions

In conclusion, the more displaced rib fractures, the more chest complications may occur. The number of displaced rib fractures could be a strong predictor for developing pulmonary complications. For patients with fewer than three rib fractures without rib displacement and initial lung or other organ injuries, outpatient management could be safe and efficient.

## Additional file

Additional file 1: Figure S1a. Displaced rib fracture in computed tomography. Arrow: Displaced rib fracture with displacement distance at least half rib width. **Figure S1b.** Displaced rib fracture in chest x ray. (DOCX 328 kb)

## Abbreviations

AIS: Abbreviated injury scale
ARDS: Acute respiratory distress syndrome
ATLS: Advanced Trauma Life Support
CI: Confidence interval
CT: Computed tomography
CXR: Chest radiograph
ICED: Index of coexistence disease
ICU: Intensive care unit
ISS: Injury severity score
NPV: Negative predictive value
OR: Odds ratio
PPV: Positive predictive value
STROBE: Strengthening the Reporting of Observational Studies in Epidemiology
TTSS: Thoracic trauma severity score
VATS: Video-assisted thoracoscopy
