# Supplementary material for: The number of displaced rib fractures is more predictive for complications in chest trauma patients
Source: Scand J Trauma Resusc Emerg Med. 2017 Feb 28;25:19. doi: 10.1186/s13049-017-0368-y (PMC5330007; doi:10.1186/s13049-017-0368-y)
Supplement: Additional file 1: Figure S1a. — Displaced rib fracture in computed tomography. Arrow: Displaced rib fracture with displacement distance at least half rib width. Figure S1b. Displaced rib fracture in chest x ray. (DOCX 328 kb) [file 13049_2017_368_MOESM1_ESM.docx]

Figure S1a Displaced rib fracture in computed tomography


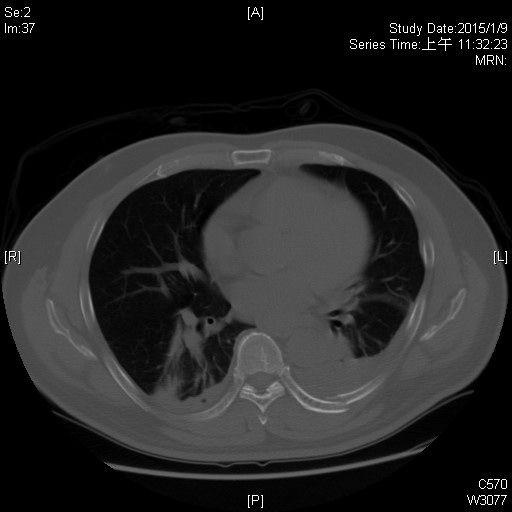


Arrow: Displaced rib fracture with displacement distance at least half rib width

Figure S1b Displaced rib fracture in chest x ray


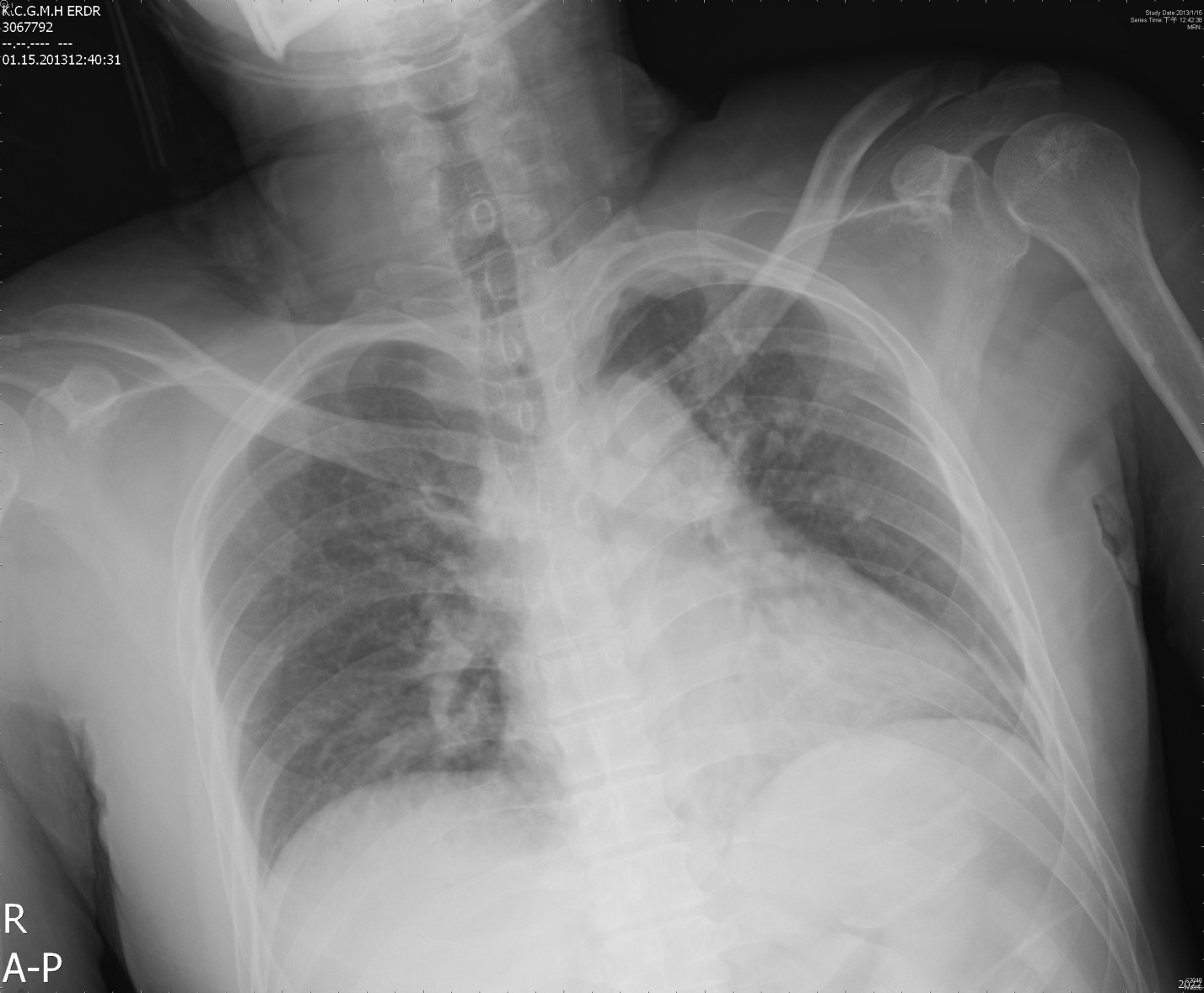


Arrow: Displaced rib fracture with displacement distance at least half rib width
